# The Influence of Vibratory Courtship on Female Mating Behaviour in Orb-Web Spiders (*Argiope keyserlingi*, Karsch 1878)

**DOI:** 10.1371/journal.pone.0053057

**Published:** 2013-01-16

**Authors:** Anne E. Wignall, Marie E. Herberstein

**Affiliations:** Department of Biological Sciences, Macquarie University, North Ryde, New South Wales, Australia; University of Western Australia, Australia

## Abstract

Web-building spiders are important models for sexual selection. While our understanding of post-copulatory mechanisms including sperm competition and cryptic female choice is considerable, our knowledge of courtship and how it influences male and female mating decisions is still extremely poor. Here, we provide the first comprehensive description of male courtship behaviour and vibrations generated in the web by the orb-web spider, *Argiope keyserlingi –* a recognised model species. We identified three main elements of male courtship: shudders, abdominal wags and mating thread dances (including both plucks and bounces). The vibrations generated by these behaviours are described in detail. Male shuddering behaviour appears to have a strong influence on female latency to mate acceptance, with males that shudder at high rates without compromising shudder duration being preferred. Shuddering behaviour may also mediate female aggressive behaviour, with males that generate long shudders less likely to be cannibalised after copulation. Male abdominal wagging behaviour, however, appears to have only limited influence on female mating decisions. This study provides avenues for future work that synthesises pre- and post-copulatory mechanisms in web-building spiders to generate an all-encompassing model of how sexual selection operates.

## Introduction

The value of spiders, particularly web-building spiders, as model systems for sexual selection is becoming increasingly evident [1], [2]. For example, one of the first pieces of evidence for cryptic female choice comes from an orb-web spider [3], [4]. Similarly, work with a range of spider species (e.g. *Linyphia*, *Latrodectus*, *Nephila* and *Argiope*) has made significant contributions to our understanding of patterns of sperm competition [5]–[9]. While we now have a detailed account of post-copulatory mechanisms in web-building spiders, we still understand very little about pre-copulatory courtship, its function, mechanism or evolution [10]. This represents a significant gap in our understanding. Courtship in many species of spiders, such as in the genus *Argiope*, often lasts an order of magnitude longer than copulation itself [11]. Therefore, courtship is of potentially significant importance for female choice and the resulting post-copulatory mechanisms.

Popular general hypotheses about the function of courtship include: stimulation of females to mate [12]–[14], species recognition [10], [14], [15], suppression of female aggression (particularly in species with sexual cannibalism [14], [15]), assessment of male quality [16] and synchronisation of mating behaviour [14]. Generating and testing functional hypotheses however requires a comprehensive description of courtship behaviour, which in web-building spiders is currently lacking.

Web-building spiders tend to have poor vision, but excellent sensitivity to vibrations [17], [18], [19]. Courting web-building spider males often engage in behaviours that involve bouncing, plucking, tugging or manipulating the silk of the female's web [14]. These behaviours generate vibrations that may provide signals or cues to the female about the presence, identity and quality of the male. To date, only a few studies have attempted to record male courtship in a female spider's web [13], [15], [20], [21], and of these only one to our knowledge has recorded courtship vibrations directly from the web (see [21]). Recordings of male funnel-web spiders were conducted using a galvonometer, a contact recording method, which may affect vibration transmission due to the weight of the stylus on the web [21]. Thus the primary aim of our study is to provide a method for the quantification and detailed descriptions of courtship vibrations in a well-studied orb-web spider. Furthermore, we identify relationships between male vibratory courtship and female behaviour as a way of developing potential functional hypotheses for future studies. In this paper we provide the first comprehensive description of the vibrations generated during courtship of male *Argiope keyserlingi* using a combination of non-contact digital laser vibrometry with synchronised video footage and high-speed video recordings. We describe in detail individual courtship elements and present preliminary evidence for how male courtship performance may influence post-copulatory processes.

## Methods

### Ethics

No specific permits were required for the described field studies. No specific permissions were required for these locations or activities. The collecting location is a public park. This location is not privately owned or protected in any way. The species used in these experiments (*Argiope keyserlingi*) is not an endangered or protected species.

### Study animals

We collected 21 sub-adult male and 21 sub-adult female *Argiope keyserlingi* from Bicentennial Park, West Pymble, Australia in October 2010. Individuals were collected as sub-adults to ensure they were virgin for our experiments, as mating status is known to influence both male and female behaviour in *A. keyserlingi* [3], [9], [22]. Males were maintained in individual, upturned plastic cups (300 mL) with a mesh top for airflow. Females were maintained in Perspex frames (50×50×10 cm) that provided space to build natural orb-webs in which we could record courtship interactions. All spiders were housed in a temperature-controlled room (25–27°C) on a 12∶12 hour Light∶Dark cycle. Females were fed weekly on crickets (*Acheta domestica*), vinegar flies (*Drosophila melanogaster*) and Queensland fruit flies (*Bactrocera tyroni*). Male spiders were fed weekly with *D. melanogaster*. All spiders were watered daily.

### Courtship and copulation in *Argiope keyserlingi*

**Figure 1:**
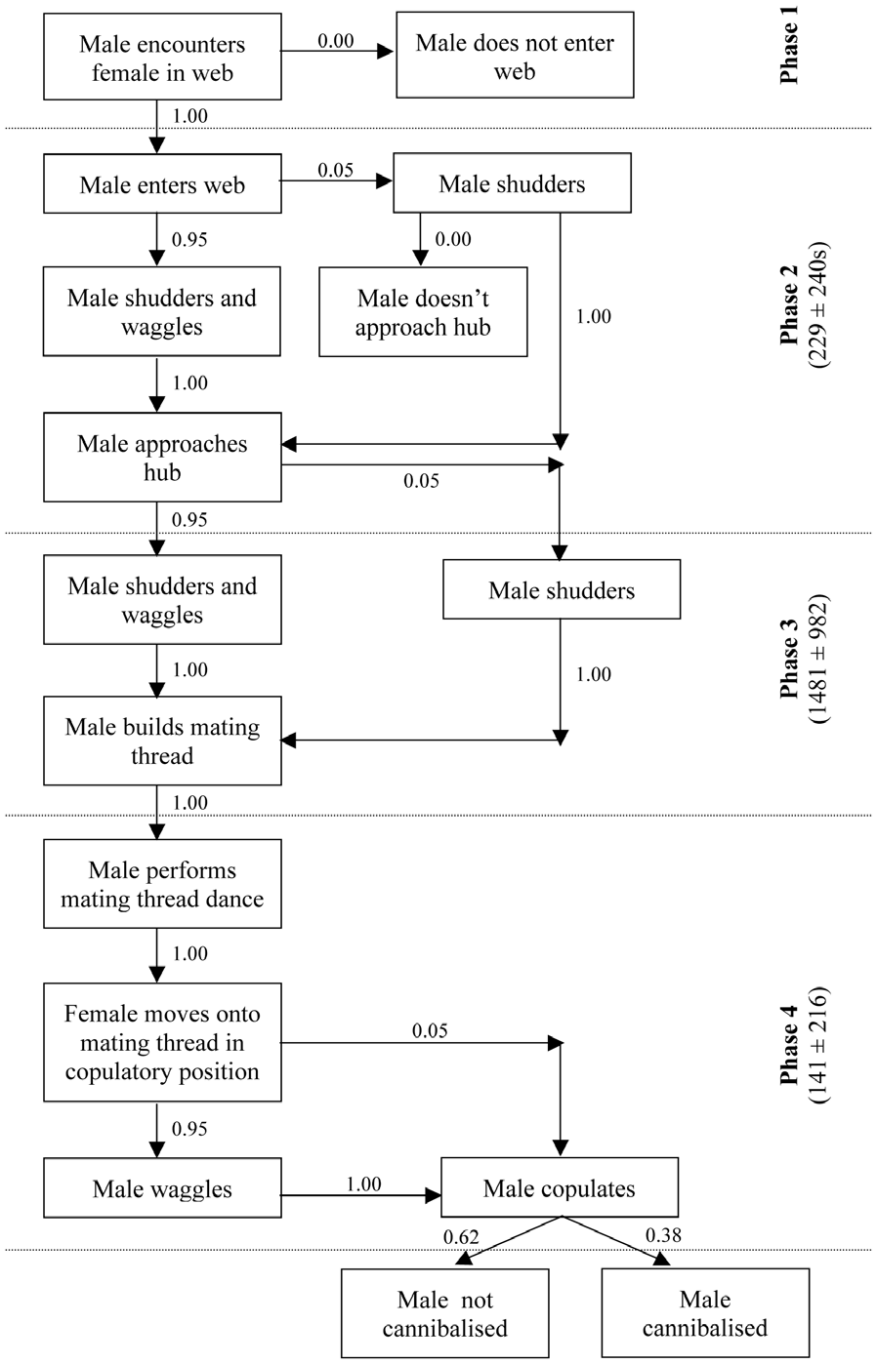
Sequence of male *A. keyserlingi* courtship. Proportions of males making transitions between each behaviour are shown beside flow arrows. Mean duration (s) (± sd) of phases 2–4 are shown.

Orb-web spider courtship (Types A, B and C) is defined according to whether courtship occurs on or off the female's web, and whether a mating thread is involved or not. Courtship in *A. keyserlingi* progresses in a fairly stereotypical manner according to ‘Type B courtship’ [14]. In Type B courtship, courtship occurs on the female's web, and males build a mating thread on which copulation occurs [14]. Male *Argiope keyserlingi* enter the female's web via the frame threads around the edges of the orb-web (Phase 1 to 2, Figure 1). The male then slowly approaches the hub at the centre of the web where the female is located (Phase 2, Figure 1). At the hub, the male ‘tastes’ the female by walking around her while contacting her legs and abdomen with his first and second pair of legs then passing his legs through his mouth (during Phase 3, Figure 1). The male spends some time at the hub engaged in this behaviour (from several minutes up to an hour or more), punctuated by short rests (often for several minutes at a time). The male then proceeds to cut out a small section of the orb web slightly above and to one side of the female at the hub. The male builds a mating thread across this space, a single line of silk that he may reinforce by laying several draglines. The male then hangs upside down from the mating thread and generates vibrations by plucking and bouncing (Phase 4, Figure 1). The female responds by moving onto the mating thread and entering a characteristic ‘copulatory position’ or ‘acceptance posture’ that exposes her genital opening, the epigynum [11], [14]. There is no evidence of copulatory courtship in *A. keyserlingi*.

### Courtship recordings

High-speed video footage of male courtship behaviours were recorded at 300 fps using a Casio Ex-F1 digital camera (Casio America, Inc., USA). Courtship vibrations were recorded using a digital laser vibrometer (Polytec PDV 100, Germany), digitized to a Digital Rapids DC 1500 A to D board using Stream 1.5.23 (Digital Rapids, Canada) recorded at 44.1 kHz/16 bits on a Windows computer (Dual 3.0 GHz Xeon, 4 GB RAM). Video footage was recorded using a 540TVL GoVideo camera (25 fps, Digital Products International Inc., USA). The AES output of the laser vibrometer was converted to EBU (CO3, Midiman, M-Audio, USA) and synchronised onto the audio track of the video. This allowed us to observe the behaviour of the spiders during the generation of vibratory stimuli. Within the web, we placed up to four *Bactrocera tyroni* wings, painted silver, at even intervals around the hub of the web. These silver wings were used as focal points for the beam of the laser vibrometer, providing a wide focal area while minimising the weight added to the web that may distort vibrations [23], [24]. This method measures transverse vibrations within the web. Longitudinal vibrations tend to travel with less attenuation than transverse vibrations in orb-webs [23]. However, measuring longitudinal vibrations using laser vibrometry is currently not possible as the movement of silk threads during male courtship is too great for the laser vibrometer's focal range. While we cannot exclude the possibility that some information about male courtship has not been captured by measuring transverse vibrations, we hope to have minimised this risk by combining our vibrational analyses with video analyses. To monitor and focus the laser vibrometer, we monitored trials using headphones connected to the laser vibrometer's analogue output from a Eurorack UB 802 soundboard (Behringer, Germany).

To commence a courtship recording, an adult male was placed at the bottom of a Perspex frame with a female in her web and allowed to make his own way into the web. If males did not enter the female's web within 30 mins, the trial was aborted. We recorded trials from when the male first made contact with female's silk, to 5 mins after copulation ended.

### Analyses

Male courtship was analysed using Adobe Soundbooth CS4 v2.0.1 (Adobe Systems Incorporated, USA), and statistical tests performed using JMP v5.0.1.2 (SAS Institute, Inc., USA). Behaviours were identified from video footage, and duration and inter-element durations were calculated from the synchronised waveform. The mean peak frequencies of individual courtship elements were calculated from a randomly selected example within an interaction. All descriptive values indicate mean ± standard deviation unless otherwise indicated. Data were checked for normal distribution (Shapiro-Wilk test) and transformed where appropriate. To estimate the relative body condition of males and females, we calculated the residuals from a regression of weight on carapace width (males: F_1, 15_ = 38.95, R^2^ = 0.72, p<0.01; females: F_1, 15_ = 13.19, R^2^ = 0.47, p<0.01) [25], [26]. Copulation success in the laboratory for virgin male – virgin female pairings is 100%, hence copulation success was not included in the analyses. While we have as yet little information on male copulation success in the field, there are indications that females are remarkably accepting of courting males [11]. We analysed male shudder and abdominal wagging behaviour for effects on female latency to move onto the mating thread (which can also be considered as latency to copulation) and copula duration, both measures that are commonly used as proxies for female preference for the male [3]. Female latency to move onto the mating thread may also be a useful proxy for female assessment of male quality due to the potential costs involved in delayed mate acceptance. Females that take longer to assess and accept a mate may reduce their prey capture rate and be at an increased risk of predation due to the presence of the male on the web [11].

We also measured the time that females spent pumping, a behaviour that indicates female disturbance in anti-predator contexts that can also be used as a measure of female ‘irritation’ during male courtship [11], [27]. Pluck rate of mating thread dances was calculated by scoring videos using JWatcher 1.0 [28]. We did not include analyses of frequency or duration for the individual elements of the mating thread dance as behaviours overlapped with each other, making individual elements difficult to analyse accurately. Sample sizes for individual analyses varied slightly according to whether a particular statistic could be calculated from each male's courtship. Our standard model for all analyses included the predictors: vibration rate, vibration duration, and the interaction between rate and duration.

## Results

Total courtship duration including time the male spent resting (defined from when the male first made contact with female silk to the start of copulation, including the mating thread dance) ranged from 11 to 96 minutes (mean = 39.51±20.78 minutes, N = 21). When we excluded male resting time, total courtship time ranged from 8 to 54 minutes (mean = 25.31±11.17 minutes, N = 21). Three main elements to male vibratory courtship were identified: shudders, abdominal wagging and mating thread dances (comprising a combination of plucks and bounces). These elements were stereotyped and easily identified. The sequence of male courtship behaviour in Figure 1 shows the probability of each transition between behaviours.

### Shudder

**Figure 2:**
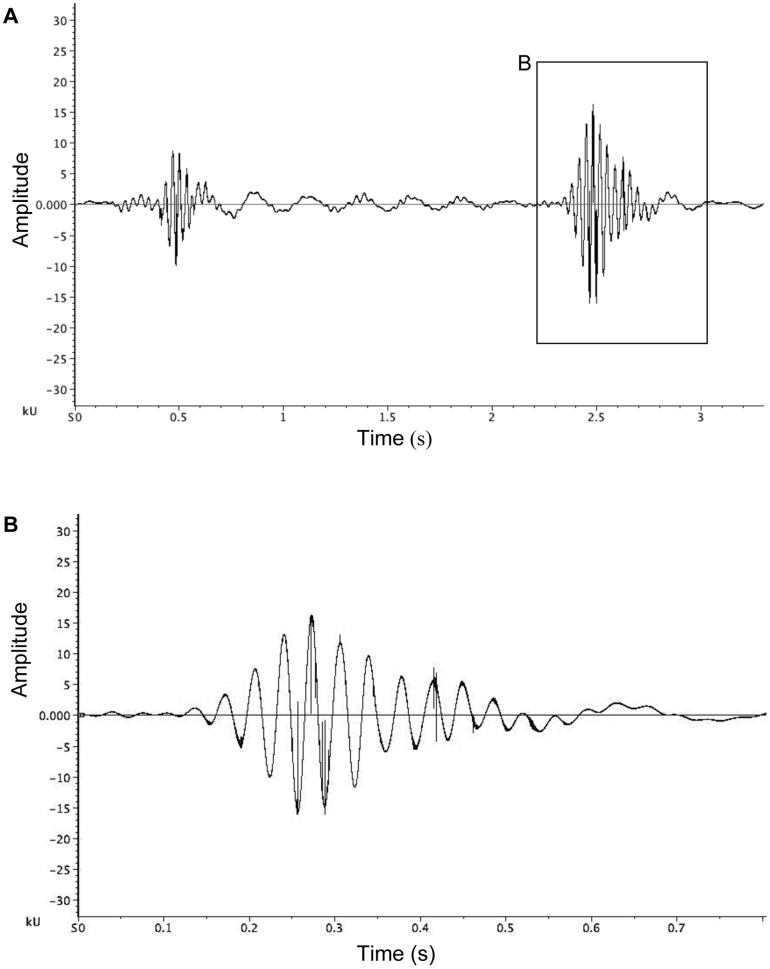
Shudder courtship vibrations of *A. keyserlingi*. (a) Waveform of a bout of shuddering by male *Argiope keyserlingi.* The apparent amplitude difference between the two shudders is due to the male approaching the female (and hence also approaching the laser vibrometer focal point), rather than a difference in courtship effort; (b) detail waveform of a single shudder from waveform (a). Shudders are performed from first contact with female silk, to commencement of courtship on the mating thread.

Shudders comprised males rocking (anterior-posterior) in the web several times, with each rock appearing to be driven by flexion of the first and second pair of legs, although this is difficult to confirm (Table 1; see high-speed video footage, Video S1). Shudders were the first vibratory courtship element to be observed within interactions. Males continued to shudder at intervals throughout the interaction until they began courtship on the mating thread (Table 1; Figure 1; Figure 2a,b). The interval between shudders was significantly shorter during the males' first approach to the female at the hub compared to the interval between shudders after reaching the hub (paired t-test: t_17_ = 4.86, p<0.01).

**Table 1:** Vibratory courtship behaviours of *Argiope keyserlingi*.

Behaviour	Description	Occurrence	Duration (s)	Inter-duration (s)	Peak Frequency (Hz)
**Shudder**	Males rock (anterior-posterior) in the web several times (4 to 12 rocks). Movement may be driven by contraction and extension of the 1st and 2nd pair of legs	Males shudder within 4.9±8.8 s (0 to 40.7 s) of coming into contact with female webs, and at intervals throughout interaction	0.42±0.06 (0.27 to 0.54)	10.95±2.89 (6.61 to 17.92)	30±9 (16 to 49)
**Abdominal wagging**	Males bob their abdomen dorso-ventrally, while the rest of their body remains stationary. Abdominal wagging occurs in bouts of several wags close together (6.67±2.08 wags per bout; range: 2.50 to 11.11)	Males abdominal wag intermittently throughout the entire interaction with a female. Males perform a bout of abdominal wagging immediately prior to a copulation attempt	0.32±0.10 (0.19 to 0.65)	Within bouts: 0.12±0.10 (0.05 to 0.51) Between bouts: 67.64±92.02 (18.68 to 446.24)	116±32 (58 to 178)
**Mating Pluck thread dance .**	Males pluck the mating thread with their 1^st^, 2^nd^ or 3^rd^ pair of legs (4^th^ pair of legs usually remain stationary on silk)	On mating thread. Dances often commence with plucks	Rate: 2.13±0.81 plucks/sec	Overlapping	
**Bounce**	Male contracts and releases legs on mating thread to bounce dorso-ventrally on thread	On mating thread. Bouncing increases as dance progresses	Total dance: 63.12±38.58 (6.73 to 128.92)	Overlapping	

All values are means ± standard deviations unless otherwise indicated. Ranges are indicated in brackets in each cell.

### Abdominal Wag

**Figure 3:**
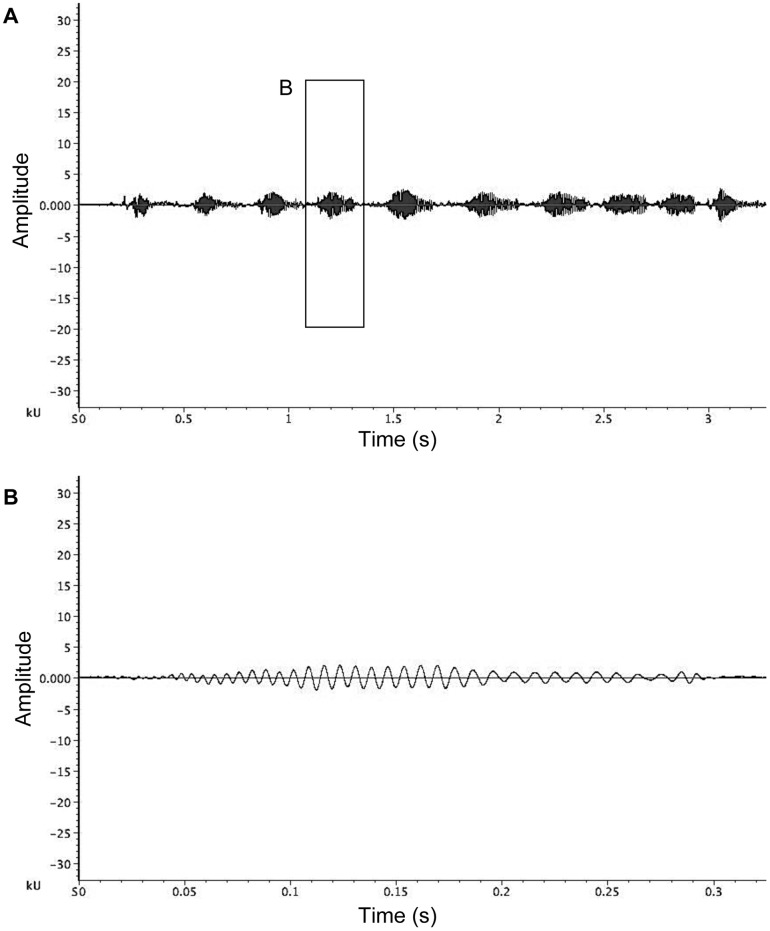
Abdominal wagging vibrations of *A. keyserlingi.* (a) Waveform of a bout of abdominal wagging by male *Argiope keyserlingi*; (b) detail waveform of a single abdominal wag from waveform (a). Abdominal wags are performed intermittently throughout courtship and immediately prior to a copulation attempt.

Abdominal wagging (as defined by [12]) comprised males rapidly bobbing their abdomen dorso-ventrally (Table 1; Video S2). Abdominal wagging tended to occur in bouts of several wags close together (Table 1; Figure 3a,b). Throughout the entire interaction, males performed on average 42.14±48.92 bouts of abdominal wags (range: 0 to 222 bouts, N = 21). Only one male never performed any abdominal wagging. All the other males performed a bout of abdominal wagging immediately prior to attempting to copulate on the mating thread (Figure 1, Phase 4).

### Male courtship behaviour and female mate choice

Total male courtship time (excluding ‘rest’ periods in which the male did not move) did not relate to female latency to move onto the mating thread (F_1,19 = _0.50, R^2^ = 0.03, p = 0.49) or copula duration (F_1,19_ = 1.22, R^2^ = 0.06, p = 0.28).

Male shuddering behaviour was significantly related to female latency to move onto the mating thread (whole model: F_3,17_ = 5.60, R^2^ = 0.50, p<0.01). The higher a male's shudder rate, the longer a female took to move onto the mating thread (shudder rate: F_1,1_ = 12.49, p<0.01), with a strong interaction between shudder rate and shudder duration (interaction: F_1,1_ = 6.64, p = 0.02). Females appeared to delay their move onto the mating thread when males with a high shudder rate consequently reduced their shudder duration.

Male shuddering behaviour did not influence copula duration (whole model: F_3, 17 = _0.76, R^2^ = 0.12, p = 0.53). However, males that generated longer shudders were less likely to be cannibalised by the female after copulation (whole model: *χ* ^2^_3_ = 10.73, p = 0.01; shudder duration: *χ* ^2^_1_ = 6.49, p = 0.01; shudder rate: *χ* ^2^_1_ = 0.23, p = 0.63; interaction: *χ* ^2^_1_ = 4.90, p = 0.03). While shudder rate alone did not seem to affect the probability of cannibalism, there was again an interaction between shudder rate and shudder duration, with males that shuddered at a higher rate decreasing their shudder duration, which increased their probability of being cannibalized. Females that pumped more were not more likely to cannibalise males (*χ* ^2^_1_ = 0.53, p = 0.47), but males took longer to begin building the mating thread when courting females that pumped more (F_1,19_ = 10.09, R^2^ = 0.35, p<0.01). However, males did not adjust their shuddering behaviour when courting females that pumped more often (whole model: F_3,17 = _0.16, R^2^ = 0.03, p = 0.92).

Male abdominal wagging did not influence the latency of females to go onto the mating thread (whole model: F_3,16_ = 2.42, R^2^ = 0.31, p = 0.10). Similarly, abdominal wagging did not influence copula duration (whole model: F_3,16_ = 0.18, R^2^ = 0.03, p = 0.91) or time the female spent pumping (F_3,16_ = 0.63, R^2^ = 0.11, p = 0.60). Similar to shuddering behaviour, males that abdominal wagged at higher rates tended to have shorter abdominal wag durations (F_1,18_ = 6.75, R^2^ = 0.27, p = 0.02). Interestingly, males that shuddered at a higher rate, tended to abdominal wag at a lower rate (F_1,18_ = 7.00, R^2^ = 0.28, p = 0.02). We found no evidence that male or female body condition correlated with male shuddering or abdominal wagging behaviour, female latency to move onto the mating thread or copula duration (Pearson correlations: all p>0.22).

### Mating thread dance

Males hung upside down from the mating thread and used their legs to strum the silk (plucks), interspersed with violent dorso-ventral and antero-posterior bouncing on the thread (bounces). Males continued to dance until the female moved onto the mating thread to adopt the copulatory position (Table 1; see Video S3). The precise sequence of the mating thread dance tended to vary between males. The mating thread dance usually commenced with several plucks by the legs on the silk thread, often the 3^rd^ pair, but sometimes also the 1^st^ or 2^nd^ pair of legs. After several plucks, the males began interspersing plucks with bounces on the mating thread (see Videos S3 and S4). Males continued to pluck and bounce on the mating thread, with the proportion of bounces tending to increase as courtship progressed. Some males commenced bouncing almost immediately on the mating thread, while others plucked for some time before the commencement of bouncing.

The mating thread dance lasted on average 63.12±38.58 seconds (range: 6.73s to 128.92s, N = 21; measured from the male's first pluck on the mating thread, to when the female was in position on the mating thread, excluding rests). If the female decided to accept the male as a mate, the female responded to the mating thread dance by approaching and hanging from the mating thread with her epigynum facing the male (all females in this study accepted the male as a mate). Sometimes it took several bouts of movement for the female to move into the correct ‘copulatory position’ on the mating thread. During these female movements, males tended to briefly cease the mating thread dance.

**Figure 4:**
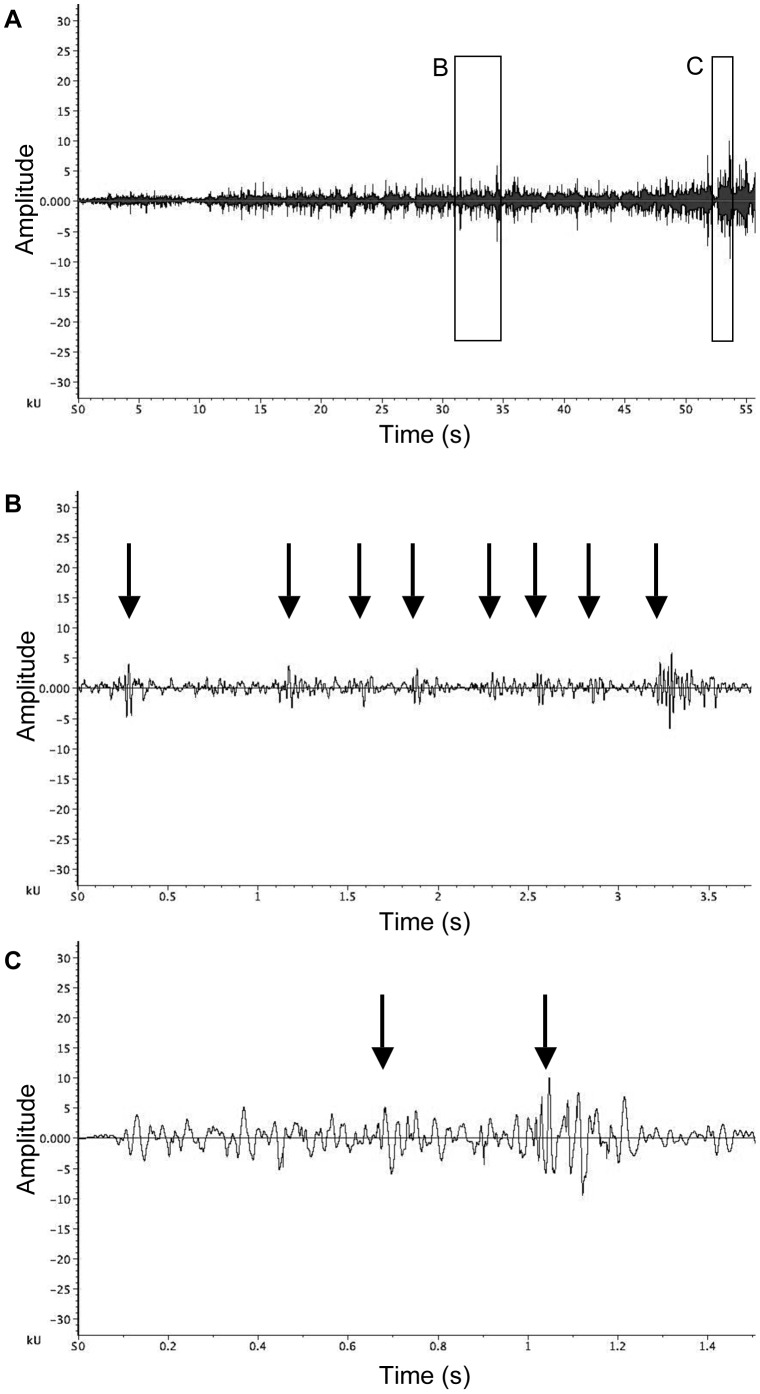
Mating thread dance vibrations of *A. keyserlingi.* (a) Waveform of the mating thread dance by male *Argiope keyserlingi*; (b) detail waveform showing several plucks from waveform (a). Some bouncing occurs simultaneously during these plucks; (c) detail waveform showing bouncing from waveform (a), including simultaneous plucks. Plucks in the waveform are indicated by arrows.

The durations of vibrations generated by individual plucks on the mating thread were not possible to calculate, due to the substantial overlap with subsequent plucks by alternate legs (Figure 4a,b). This also interfered with calculation of the interval between plucks. Similarly, bounces were difficult to analyse as they also overlapped with plucks (Figure 4c). The amplitude of vibrations generated during the mating thread dance tended to increase as courtship progressed (Figure 4a).

## Discussion

Female preference for a mate in orb-web spiders is often measured by latency to move onto the mating thread (as an indication of willingness to mate) and copula duration (under female control, with the potential to limit the amount of sperm males can transfer) [3], [11]. Our results indicate that the duration of male courtship time alone does not influence female preference behaviour. This appears to contrast with *Argiope bruennichi*, in which males with a short courtship duration had a significantly reduced paternity share compared to males that courted for longer durations [29]. Male redback spiders, *Lactrodectus hasselti*, with short courtship durations also have lower paternity compared to males with longer courtship durations as females prematurely cannibalise males that invest little in courtship duration [30], [31]. While our study did not assess paternity in relation to male courtship effort, our results suggest that, in *A. keyserlingi*, individual elements of courtship quality, rather than courtship duration *per se*, influences female choice behaviours.

Male shuddering behaviour appears to have a strong influence on female latency to mate acceptance. The higher the male's shudder rate, the longer a female will take to move into the characteristic acceptance posture on the mating thread. This appears to be due to males with very high shudder rates compromising their ability to generate shudders of a ‘reasonable’ duration. It is unlikely that males increase their shudder rate (and hence reduce their shudder duration) in response to female reluctance to mate, as female reluctance is mostly assessed by the male once on the mating thread (Phase 4), and not during Phases 2–3 (see Figure 1). Female reluctance to mate is exhibited by either a long latency to move onto the mating thread, or possibly through aggressive behaviours such as pumping. However, our results show that males do not appear to adjust their shuddering behaviour when courting highly aggressive females. While shudder duration *per se* does not appear to influence female assessment of males, it appears that females will take longer to assess and accept as a mate those males that allow their shudder duration to drop below a threshold level when shuddering at higher rates.

Courtship and copulation can be very dangerous for web-building spiders, with many females from diverse species attacking and often cannibalising males before, during or after copulation [32]–[35]. It has been suggested in several spider species that courtship displays may help lower female aggression [20], [36], [37]. In *A. keyserlingi*, male courtship was not directly influenced by female aggressive pumping behaviour. However, males that courted females that pumped at a high rate took longer to begin building the mating thread. It would be interesting to discover whether males increase their courtship duration (Phases 1–3, Figure 1) to ameliorate the risk of cannibalism by these females. Male shuddering behaviour may also be important in regulating female aggressive behaviours after copulation. Males with longer shudders appear to have a reduced risk of post-copulatory cannibalism, which in turn increases their potential for a second mating and higher reproductive fitness than males with shorter shudders (that shudder at higher rates).

Male abdominal wagging appears to have only limited influence on female latency to move onto the mating thread, copula duration and pumping behaviour. However, abdominal wagging is a common element of spider courtship behaviour, and has been observed in diverse spider families [20], [37]–[39]. Interestingly, all males that performed abdominal wagging completed a bout immediately prior to approaching the female for a copulation attempt. One potential function of abdominal wagging may be to increase haemolymph pressure in the pedipalps prior to insertion for sperm transfer [40]. Therefore, while this behaviour is performed during the courtship phase, it may have no function in female mate assessment.

There is little evidence from the current study to suggest that male courtship effort is condition dependent. However, males were maintained in similar laboratory conditions under a high quality diet, which may provide only limited scope for investigation of condition-dependent signalling behaviours. Previous work on wolf spiders has indicated that male courtship vibrations vary when male diet is manipulated during development [41]–[43]. This provides an avenue for future research concentrating on diet manipulations of individual males through development and their effects on courtship behaviour and female preference.

Understanding courtship behaviour in male orb-web spiders such as *A. keyserlingi* provides a unique opportunity to formulate a cohesive theory underlying the evolution of mating systems. A wealth of information has been collected about post-copulatory mechanisms in *A. keyserlingi* and closely related species. However, how courtship informs mating decisions and hence influences reproductive fitness has until now remained elusive. This study will help open up future research incorporating pre- and post-copulatory mechanisms into sexual selection models.

## Supporting Information

Video S1 **High-speed (300 fps) footage of a male shuddering.**
(MOV)

Video S2 **Male abdominal wagging.**
(MOV)

Video S3 **Start of male mating thread dance, showing plucks and bounces.**
(MOV)

Video S4 **High-speed (300 fps) footage of male mating thread dance, showing plucks and bounces.**
(MOV)
